# Antimicrobial susceptibility profile of oral and rectal microbiota of non‐human primate species in Ghana: A threat to human health

**DOI:** 10.1002/vms3.1271

**Published:** 2023-09-21

**Authors:** Eugene Adade, Patrick Ofori Tawiah, Christian Roos, Idrissa Shomari Chuma, Clara Clavery Lubinza, Sayoki Godfrey Mrinde Mfinanga, Sascha Knauf, Augustina Angelina Sylverken

**Affiliations:** ^1^ Department of Theoretical and Applied Biology Kwame Nkrumah University of Science and Technology Kumasi Ghana; ^2^ Kumasi Centre for Collaborative Research in Tropical Medicine Kwame Nkrumah University of Science and Technology Kumasi Ghana; ^3^ Gene Bank of Primates and Primate Genetics Laboratory German Primate Center Leibniz Institute for Primate Research Göttingen Germany; ^4^ Tanzania National Parks Arusha Tanzania; ^5^ National Institute for Medical Research Muhimbili Medical Research Centre Dar es Salaam Tanzania; ^6^ Institute of International Animal Health/One Health Friedrich‐Loeffler‐Institut Federal Institute for Animal Health Greifswald – Insel Riems Germany

**Keywords:** antimicrobial resistance, public health, wildlife conservation, wildlife, zoonotic bacteria, zoonotic transmission

## Abstract

**Background:**

The potential for the transfer of zoonotic diseases, including bacteria between human and non‐human primates (NHPs), is expected to rise. It is posited that NHPs that live in close contact with humans serve as sentinels and reservoirs for antibiotic‐resistant bacteria.

**Objectives:**

The objective was to characterize the oral and rectal bacteria in Ghanaian NHPs and profile the antimicrobial susceptibility of the isolated bacteria.

**Methods:**

Oral and rectal swabs were obtained from 40 immobilized wild and captive NHPs from 7 locations in Ghana. Standard bacteriological procedures were used in the isolation, preliminary identification, automated characterization and antimicrobial susceptibility test (AST) of bacteria using the Vitek 2 Compact system.

**Results:**

Gram‐negative bacteria dominated isolates from the rectal swabs (*n* = 76, 85.4%), whereas Gram‐positive bacteria were more common in the oral swabs (*n* = 41, 82%). *Staphylococcus haemolyticus* (*n* = 7, 14%) was the most occurring bacterial species isolated from the oral swabs, whereas *Escherichia coli* (*n* = 32, 36%) dominated bacteria isolates from rectal swabs. *Enterobacter* spp. had the highest (39%) average phenotypic resistance to antimicrobials that were used for AST, whereas a trend of high resistance was recorded against norfloxacin, Ampicillin and Tetracycline in Gram‐negative bacteria. Similarly, among Gram‐positive bacteria, *Staphylococcus* spp. had the highest (25%) average phenotypic resistance to antimicrobials used for AST, and a trend of high resistance was recorded against penicillin G and oxacillin.

**Conclusions:**

This study has established that apparently healthy NHPs that live in anthropized environments in Ghana harbour zoonotic and antimicrobial resistant bacteria.

## Graphical abstract

The study showed that apparently healthy non‐human primates in Ghana harbour zoonotic antimicrobial‐resistant bacteria: pathogenic bacteria, such as *Escherichia coli*, *Enterobacter* spp., *Klebsiella* spp., *Proteus* spp., *Enterococcus* spp. and *Staphylococcus* spp. The bacteria isolated from the non‐human primates were resistant to commonly used antibiotics in humans and animals, such as Norfloxacin, Ampicillin, Tetracycline, penicillin G, and oxacillin.

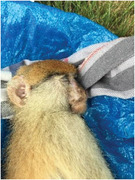

## 1 INTRODUCTION

Although wildlife is generally not the driver for pandemics, it is known to serve as a constant source for microorganisms that have the potential to cause pandemics and panzootics (Can et al., 2019). In particular, pathogens that are transmitted among wildlife, livestock and humans pose a challenge to wildlife conservation and threat to human and animal health (Rwego et al., 2008). Over the years, the contact between wild non‐human primates (NHPs) and human in Africa has intensified. Some of the reasons for this are human encroachment and resulting habitat destruction and (eco‐)tourism (Rwego et al., 2008). The close relationship between NHPs and human further increases the potential for the exchange of zoonotic pathogens (Vore et al., 2001).

Antimicrobial resistance (AMR) is a pandemic that is of global concern. It goes largely unrecognized even though 700,000 people die from infection with AMR annually (Pokharel et al., 2020). Although the One Health approach is more and more accepted, there is still inadequate cross‐sectorial collaboration with regard to AMR (O'Neill, 2016; Pokharel et al., 2020).

Isolation of potentially zoonotic pathogenic bacteria in apparently healthy NHPs has been reported in West Africa (Okwori et al., 2014, 2013). The number of transmission routes, such as faecal–oral or through bites (Devaux et al., 2019) highlights the risk of the exchange of pathogenic bacteria between human and NHPs.

There are indications that bacteria recovered from bite wounds are generally reflective of the oral bacteria of the biting animal. This is particularly significant in human–NHP interactions as it has been reported that NHPs that have close contact with humans usually inflict bite wounds (Abrahamian & Goldstein, 2011). A number of pathogenic bacteria, such as *Enterobacteria*, *Staphylococcus* spp., *Streptococcus* spp., *Neisseria* spp. and *Haemophilus* spp. have been isolated from the oral cavity of NHPs (Devaux et al., 2019; Sobreira et al., 2019). With regards to the faecal–oral route of transmission, apparently healthy NHPs have been reported to harbour pathogenic enteric bacteria, such as *Escherichia coli*, *Klebsiella* spp., *Salmonella* spp., *Shigella* spp. and *Campylobacter* spp. (Devaux et al., 2019). NHPs and humans are generally susceptible to infections caused by pathogenic enteric bacteria through zoonotic and anthroponotic transmissions even though they can be asymptomatic carriers (Medkour et al., 2021; Okwori et al., 2014).

Proximity of NHPs to human or anthropized environments can facilitate the transfer of antibiotic‐resistant bacteria (ARB) between human and NHPs. This is significant as wild animals, such as NHPs with the exception of captive animals, usually do not come into contact with antimicrobials (Parsons et al., 2021). The exchange of ARB between human and NHPs in close proximity threatens not only human health but also animal health (Albuquerque et al., 2020). Given the reports of apparently healthy NHPs harbouring zoonotic bacteria pathogens (Egbetade et al., 2020; Okwori et al., 2014, 2013), it becomes important to conduct surveillance on the oral and rectal bacteria and their antimicrobial susceptibility profile of NHPs to provide information relevant to public health and NHP conservation. In this study, we therefore characterized the oral and rectal bacteria of NHPs in Ghana and determined their antimicrobial susceptibility profile.

## 2 MATERIALS AND METHODS

### 2.1 Study design

This is a cross‐sectional study which involved NHPs in Ghana that have high interaction with humans. Permits were obtained from the Wildlife Division of the Forestry Commission of Ghana with number 0095189 for the sampling of NHPs according to their approved guidelines. Good veterinary practice rules were applied to all sampling procedures, and all samples were taken by a licensed veterinarian.

### 2.2 Study areas

**FIGURE 1:**
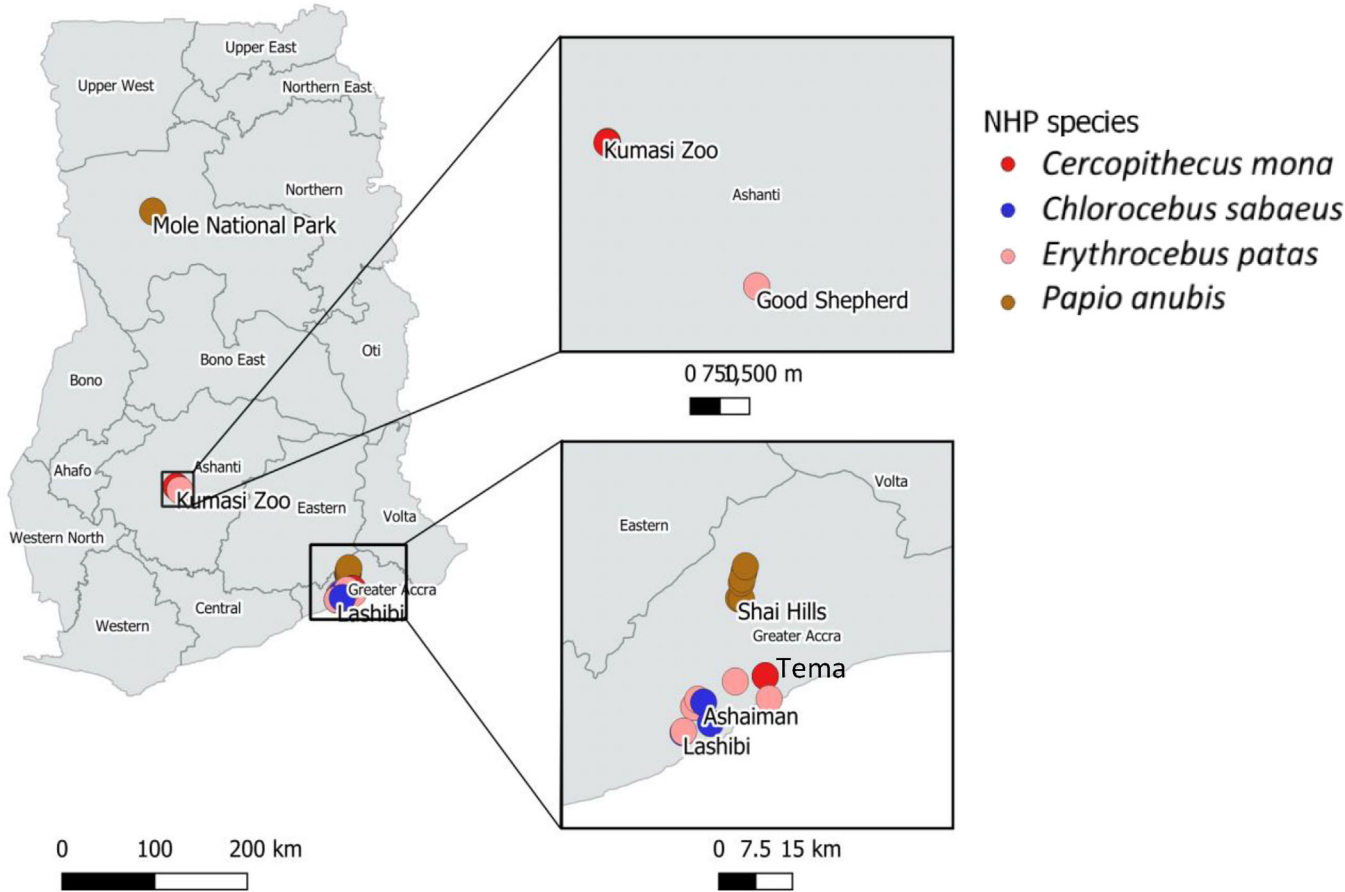
A map showing the sample locations generated with ArcGIS version 3.8.2. *Papio anubis* were the only non‐human primate (NHP) species sampled in Mole National Park in the Savannah Region; *Cercopithecus mona* and *Erythrocebus patas* were the NHP species sampled in the Ashanti Region in Kumasi Zoo. All four NHP species were found and sampled in Tema and its environs in the Greater Accra Region.

We sought information from the Ghana Wildlife Division on possible areas with high NHP–human contact. With this, the study areas were purposively selected based on the level of NHP‐human contact. The sample locations for NHPs in this study were Mole National Park (Savannah Region), Shai Hills Resource Reserve (Greater Accra Region), Kumasi Zoological Gardens (Ashanti Region), Good Shepherd Preparatory School (Ashanti Region), Tema (Greater Accra Region), Lashibi (Greater Accra Region) and Ashaiman (Greater Accra Region) (Figure 1). The NHPs sampled in Mole National Park had frequent contact with humans at the residence of park staff. The NHPs sampled in Shai Hills Resource Reserve were at the entrance area of the reserve and had frequent contact with tourists. The NHPs from Kumasi Zoo were confiscated from individuals who kept them illegally. The NHPs from Good Shepherd Preparatory School, Tema, Ashaiman and Lashibi were kept illegally as pets.

### 2.3 Number and species of non‐human primates captured

The captured NHPs represent four catarrhine species: *Erythrocebus patas* (*n* = 17, 42.5%), *Papio anubis* (*n* = 15, 37.5%), *Chlorocebus sabaeus* (*n* = 5, 12.5%) and *Cercopithecus mona* (*n* = 3, 7.5%; Table 1). The 40 sampled individuals comprise 21 males and 19 females. According to dentition, individuals were categorized into different age classes (adult, sub‐adult and juvenile) with a total of 17 adults, 11 sub‐adults and 12 juveniles. Further details can be found in Table 1.

**TABLE 1:** Detailed information about sampled non‐human primates.

NHP species	No. (%)	No. (%) male	No. (%) female	No. (%) adult	No. (%) sub‐adult	No. (%) juvenile
*Erythrocebus patas*	17 (42.5)	5 (23.8)	12 (63.2)	4 (23.5)	7 (63.6)	6 (50.0)
*Papio anubis*	15 (37.5)	11 (52.4)	4 (21.1)	9 (52.9)	3 (27.3)	3 (25.0)
*Chlorocebus sabaeus*	5 (12.5)	5 (23.8)	0	2 (11.8)	1 (9.1)	2 (16.7)
*Cercopithecus mona*	3 (7.5)	0	3 (15.8)	2 (11.8)	0	1 (8.3)
Total	40 (100)	21 (100)	19 (100)	17 (100)	11 (100)	12 (100)

Abbreviation: NHP, non‐human primate.

### 2.4 Sampling of non‐human primates

Forty NHPs were chemically immobilized by remote distance injection with a combination of Ketamine (Ketavet, Pfizer) (10 mg/kg body weight) and medetomidine (Domitor, Pfizer) (0.2 mg/kg body weight). Immobilized NHPs were first weighed with a scale (KERNCH) before being placed on the dorsal recumbency for sampling to commence. A physical examination of the NHPs was done, and vital parameters, such as body temperature, respiratory rate, heart rate and blood oxygen saturation, were taken every 5 min during sampling. The pulse, heart rate and blood oxygen saturation were monitored with a pulse oximeter (OxiMax N65, Nellcor). One oral (FITZCO CEP swabs) and one rectal (Polyester‐tipped swabs) swab samples were obtained aseptically from the oral cavity and rectum of each of the animals, respectively. The samples were transported on ice to the laboratories of the Kumasi Centre for Collaborative Research in Tropical Medicine (KCCR) for subsequent analysis.

### 2.5 Culture of swab samples

The samples were subjected to aerobic culture at 37°C for 18–24 h. Oral swabs were cultured on Columbia blood (BD Difco), MacConkey (BD Difco) and Chocolate (BD Difco) agars. Rectal swabs were cultured on Columbia blood, MacConkey and xylose lysine deoxycholate (XLD) (Oxoid) agars. Bacteria isolated from swab samples were initially characterized morphologically and biochemically and Gram‐stained. Isolates of all bacteria were stored at −20°C in brain heart infusion (BHI) broth (BD BBL) with 20% glycerol.

### 2.6 Automated bacteria identification

Polystyrene tubes (12 mm × 75 mm) (Sarstedt) were filled with 3 mL of 0.45% sterile saline (CareFusion). Homogenous suspensions of bacteria were prepared in the 3 mL 0.45% sterile saline. Suspensions were examined using the DensiCheck Plus (BioMérieux) device to ensure that the suspensions were within the turbidity range of 0.5–0.63 (McFarland standard) for both Gram‐positive and Gram‐negative bacteria. The appropriate Vitek cards (Vitek GP and Vitek GN) (BioMérieux), which contained 64 wells with miniaturized selective media and biochemical reagents, were used for bacterial identification. The Vitek system identified the bacteria by filling the Vitek wells with the standard turbidity bacteria suspension in 3 mL of 0.45% sterile saline. The colour codes from the miniaturized selective media as well as the results of the miniaturized biochemical tests were analysed by the Vitek 2 Compact system software (Version 08.01, BioMérieux) to identify the bacteria species.

### 2.7 Automated antimicrobial susceptibility test

Polystyrene tubes (12 mm × 75 mm) were filled with 3 mL 0.45% sterile saline. A volume of 145 and 280 μL of McFarland turbidity standard of homogenized bacteria suspension in 3 mL 0.45% sterile saline was pipetted into the polystyrene tubes for Gram‐positive and Gram‐negative ASTs, respectively. AST results were obtained after the Vitek system had analysed the minimum inhibitory concentration of the bacterial species against the prescribed antibiotics of the Vitek AST cards (AST‐N and AST‐P) (BioMérieux). The antibiotics included in the AST‐N cards were Cefuroxime, Ampicillin, Ciprofloxacin, Tetracycline, Trimethoprim–Sulfamethoxazole, Amoxicillin/clavulanic acid, Gentamicin, Meropenem, Ceftazidime, Norfloxacin and Nitrofurantoin. The antibiotics included in the AST‐P cards were Vancomycin, Clindamycin, Ciprofloxacin, Tetracycline, Trimethoprim–Sulfamethoxazole, Erythromycin, Gentamicin, penicillin G and oxacillin.

### 2.8 Data analysis

Data on the captured NHPs and characterized bacteria with AST were entered into Microsoft Office Excel 2019. Descriptive analyses were performed to generate the proportion of bacteria populations, and the frequency and distribution of Gram‐positive, Gram‐negative, resistant or sensitive bacteria were analysed with Microsoft Office Excel 2019 and Graph Pad Prism (Version 8). Figures were generated with Adobe Illustrator 2022. Chi‐square test was used to determine whether there was statistical difference between antimicrobial susceptibility profile and the body part (oral cavity and rectal region) from which bacteria were isolated, as well as the sex and origin (wild and captive) of NHPs. Any result with a *p‐*valu*e* ≤0.05 was considered statistically significant.

## 3 RESULTS

### 3.1 Diversity of bacteria isolates

**FIGURE 2:**
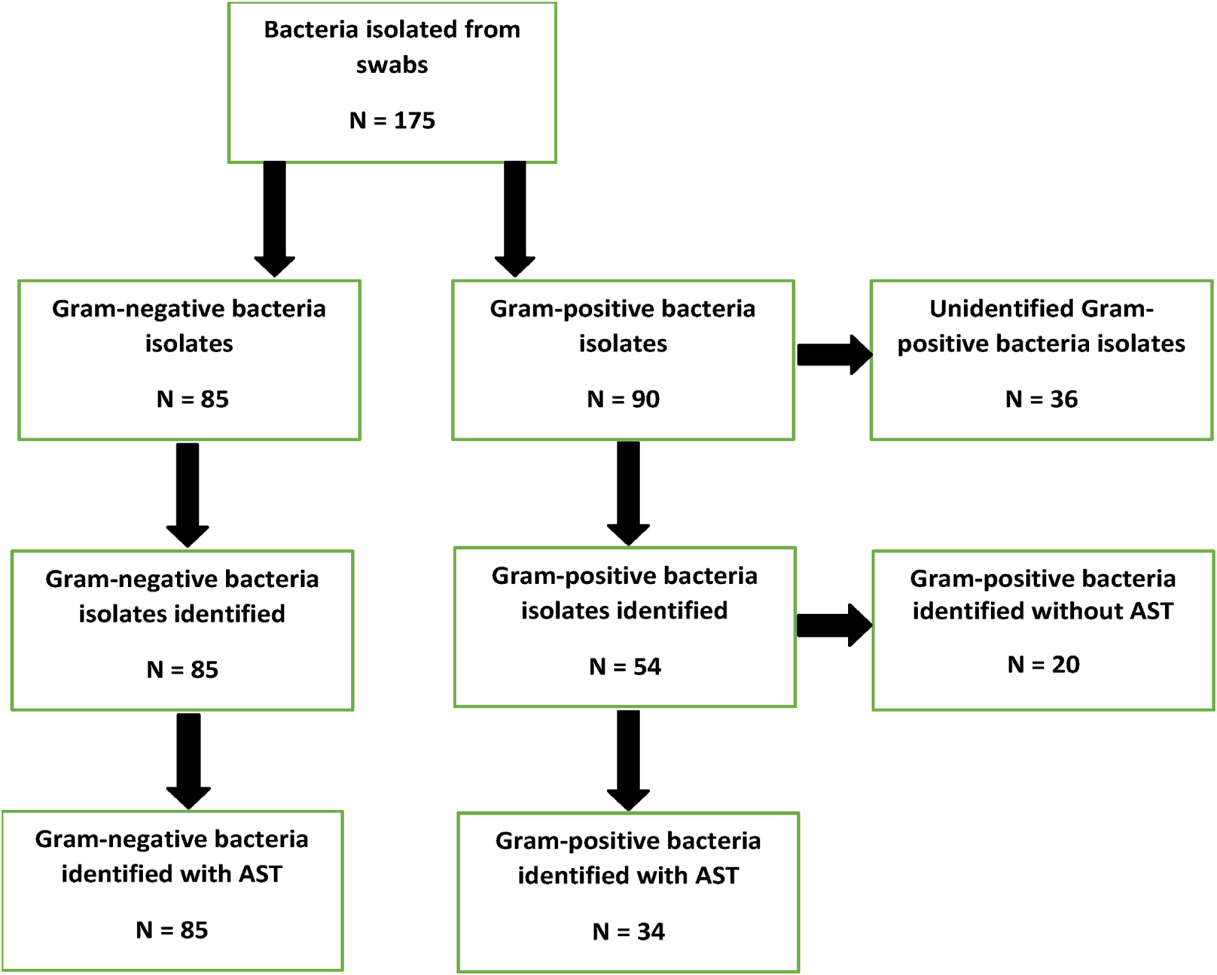
Distribution of bacteria isolated from the oral and rectal swabs. Out of 175 bacteria isolates, 85 isolates were Gram‐negative and 90 were Gram‐positive. Out of the 90 Gram‐positive bacteria, 36 were unidentified, whereas 20 of the remaining 54 Gram‐positive isolates were identified by the Vitek 2 system without an automated antimicrobial susceptibility test (AST). There were 85 Gram‐negative and 34 Gram‐positive isolates that had their automated identification and AST performed by the Vitek 2 system.

A total of 175 bacterial isolates were isolated from the oral and rectal swabs. Of this, 36 (20.6%) could not be identified by the Vitek 2 system. Of the remaining 139 isolates, 85 (61.2%) and 54 (38.8%) were identified as Gram‐negative and Gram‐positive bacteria, respectively. Out of these 139 isolates, automated identification and AST was achieved for 119 (85.6%) bacterial isolates. The 119 isolates were composed of 85 (71.4%) Gram‐negative bacteria and 34 (28.6%) Gram‐positive bacteria. All these isolates had their antimicrobial susceptibility profile performed. A summary of the distribution of bacteria isolated from the oral and rectal swabs is shown in Figure 2.

Out of the total bacteria isolated from the rectal swabs, 76 (85.4%) were Gram‐negative bacteria, whereas 13 (14.6%) were Gram‐positive bacteria. However, from the oral swabs, 41 (82%) of the isolates were Gram‐positive bacteria and 9 (18%) were Gram‐negative. Overall, 139 isolates from 15 genera were identified from the oral and rectal swabs. *Staphylococcus* spp. were the most prevalent bacteria isolated from the oral swabs (*Staphylococcus haemolyticus*: *n* = 7, 14%; *Staphylococcus gallinarum*: *n* = 6, 12%; *Staphylococcus sciuri*: *n* = 6, 12%). *E. coli* (*n* = 32, 36%) and *Klebsiella pneumoniae* ssp*. pneumoniae* (*n* = 16, 18%) were the most occurring bacteria isolated from rectal swabs (Table 2).

**TABLE 2:** Distribution of identified bacteria species.

		Oral swabs	Rectal swabs	Total
Class of bacteria	Bacterial species	*n* = 50 (%)	*n* = 89 (%)	*n* = 139 (%)
**Gram‐positives**	*Streptococcus sanguinis*	1 (2.0)	0 (0.0)	1 (0.7)
	*Streptococcus pluranimalium*	1 (2.0)	0 (0.0)	1 (0.7)
	*Streptococcus parasanguinis*	1 (2.0)	0 (0.0)	1 (0.7)
	*Streptococcus mitis/oralis*	1 (2.0)	0 (0.0)	1 (0.7)
	*Staphylococcus xylosus*	1 (2.0)	0 (0.0)	1 (0.7)
	*Staphylococcus warneri*	1 (2.0)	1 (1.1)	2 (1.4)
	*Staphylococcus sciuri*	6 (12.0)	0 (0.0)	6 (4.3)
	*Staphylococcus pseudintermedius*	1 (2.0)	0 (0.0)	1 (0.7)
	*Staphylococcus kloosii*	2 (4.0)	0 (0.0)	2 (1.4)
	*Staphylococcus hominis* spp. *hominis*	1 (2.0)	0 (0.0)	1 (0.7)
	*Staphylococcus haemolyticus*	7 (14.0)	1 (1.1)	8 (5.8)
	*Staphylococcus gallinarum*	6 (12.0)	0 (0.0)	6 (4.3)
	*Staphylococcus auricularis*	1 (2.0)	0 (0.0)	1 (0.7)
	*Staphylococcus aureus*	1 (2.0)	2 (2.2)	3 (2.2)
	*Staphylococcus arlettae*	3 (6.0)	0 (0.0)	3 (2.2)
	*Rothia dentocariosa*	2 (4.0)	0 (0.0)	2 (1.4)
	*Leuconostoc mesenteroides* spp. *cremoris*	1 (2.0)	0 (0.0)	1 (0.7)
	*Granulicatella adiacens*	1 (2.0)	0 (0.0)	1 (0.7)
	*Gemella morbillorum*	1 (2.0)	0 (0.0)	1 (0.7)
	*Enterococcus hirae*	0 (0.0)	4 (4.5)	4 (2.9)
	*Enterococcus gallinarum*	1 (2.0)	0 (0.0)	1 (0.7)
	*Enterococcus faecalis*	0 (0.0)	3 (3.4)	3 (2.2)
	*Enterococcus durans*	0 (0.0)	2 (2.2)	2 (1.4)
	*Dermacoccus nishinomiyaensis*	1 (2.0)	0 (0.0)	1 (0.7)
**Gram‐negatives**	*Acinetobacter baumannii complex*	0 (0.0)	1 (1.1)	1 (0.7)
	*Citrobacter amalonaticus*	0 (0.0)	1 (1.1)	1 (0.7)
	*Enterobacter cloacae complex*	1 (2.0)	12 (13.5)	13 (9.4)
	*Enterobacter aerogenes*	0 (0.0)	2 (2.2)	2 (1.4)
	*E. cloacae* spp. *cloacae*	1 (2.0)	0 (0.0)	1 (0.7)
	*Escherichia coli*	3 (6.0)	32 (36.0)	34 (24.5)
	*Klebsiella pneumoniae* spp. *pneumoniae*	3 (6.0)	16 (18.0)	19 (13.7)
	*Klebsiella oxytoca*	0 (0.0)	1 (1.1)	1 (0.7)
	*Proteus mirabilis*	0 (0.0)	11 (12.4)	11 (7.9)
	*Providencia stuartii*	1 (2.0)	0 (0.0)	1 (0.7)

### 3.2 Proportion of bacteria species harboured by non‐human primates

**FIGURE 3:**
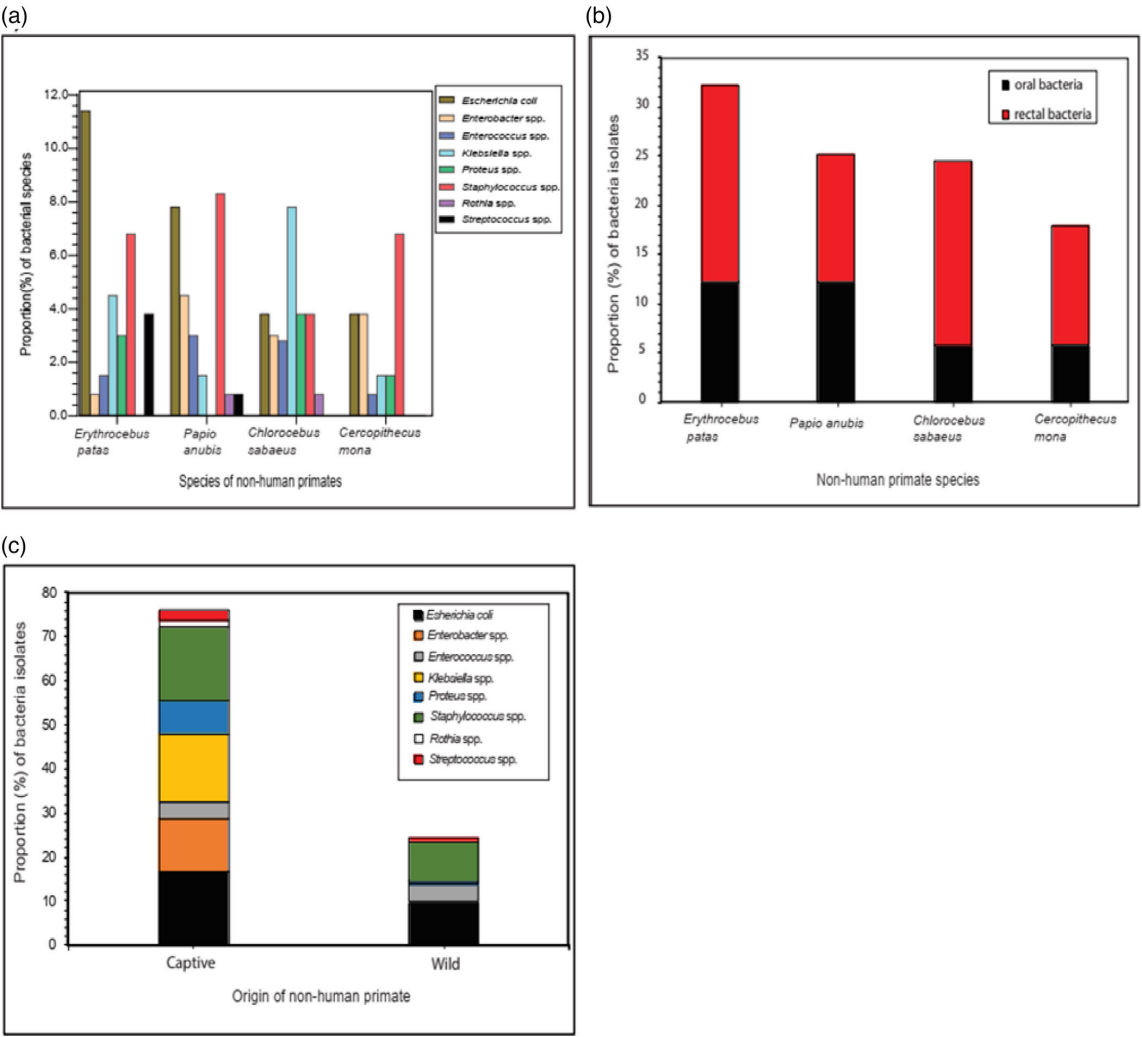
Proportion of bacterial species (**a) based on bacterial proportion per non‐human primate (NHP)**: *Escherichia coli* was the most abundant species in *Erythrocebus patas*, whereas *Staphylococcus* spp. were the dominant isolates in both *Papio anubis* and *Cercopithecus mona*. *Klebsiella* spp. were the dominant species in *Chlorocebus sabaeus*; **(b) based on anatomical origin**: *E. patas* harboured the highest proportion of rectal bacterial isolates, whereas both *E. patas* and *P. anubis* had the highest oral bacterial isolates; (**c) based on origin**: *Enterobacter* spp., *Klebsiella* spp. and *Rothia* spp. were absent in isolates from wild NHPs.

The 40 NHPs which were included into the study harboured bacteria in the oral and rectal region in various proportions; in *E. patas*, *E. coli* (*n* = 15, 11.4%) was the most prevalent bacterium isolated. *Staphylococcus* spp. were the dominant isolates in both *P. anubis* and *C. mona*. *Klebsiella* spp. (*n* = 10, 7.8%) also dominated in *C. sabaeus* (Figure 3a). In the distribution of oral and rectal bacterial isolates, *E. patas* had the highest proportion (*n* = 28, 20.1%) of rectal bacterial isolates followed by *C. sabaeus* (*n* = 26, 18.7%). Both *P. anubis* and *E. patas* had the highest proportion (*n* = 17, 12.2%) of oral bacterial isolates. Across all the NHP species, there was a higher proportion of rectal bacterial isolates compared to oral bacterial isolates (Figure 3b). More bacterial species were isolated from the oral and rectal regions of captive NHPs compared to wild NHPs. Isolates of *Enterobacter* spp., *Klebsiella* spp. and *Rothia* spp. were absent in the cultured samples of wild NHPs (Figure 3c).

### 3.3 Antimicrobial susceptibility profile of isolated bacteria

**FIGURE 4:**
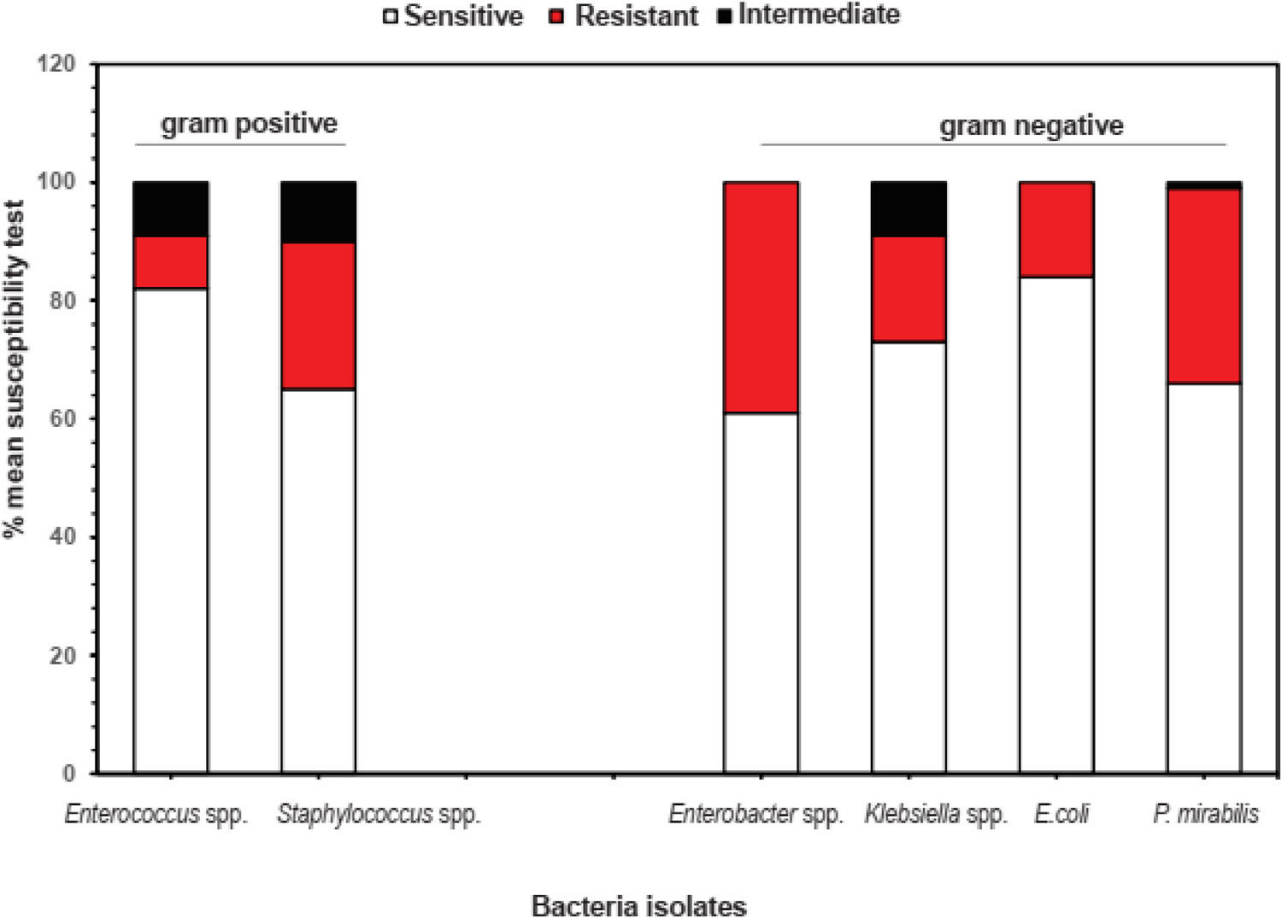
Antimicrobial susceptibility profile of species of Gram‐positive and Gram‐negative bacteria. Black, red and white were used to represent intermediate, resistant and susceptible proportions, respectively, in the stacked bar graph. *Staphylococcus* spp. had the highest average phenotypic resistance against antibiotics used for Gram‐positive bacteria, whereas *Enterococcus* spp. was most sensitive to the antibiotics (left). *Enterobacter* spp. had the highest average phenotypic resistance against antibiotics used for Gram‐negative bacteria, whereas *Escherichia coli* was most sensitive to the antibiotics (right).

Automated AST was performed by Vitek 2 employing the AST‐N and AST‐P cards. The results were recorded according to the European Committee on Antimicrobial Susceptibility Testing (EUCAST) guidelines. *Staphylococcus* spp. recorded the highest average phenotypic resistance (25%) against the antibiotics used for Gram‐positive bacteria, whereas *Enterococcus* spp. recorded an average of 82% sensitivity to the antibiotics. *Enterobacter* spp. recorded the highest average phenotypic resistance (39%) against antibiotics used for Gram‐negative bacteria, whereas *E. coli* recorded an average of 84% sensitivity to the same antibiotics (Figure 4).

Among the 85 Gram‐negative bacterial isolates, the highest frequency of phenotypic resistance was found against Norfloxacin (98%) followed by Ampicillin (56%) and Tetracycline (36%), and a high frequency of sensitivity to Meropenem (100%), Ciprofloxacin (99%) and Gentamicin (99%) (Table 3). Among the 34 Gram‐positive bacterial isolates, the highest frequency of phenotypic resistance was against penicillin G (56%) followed by oxacillin (35%), and a high frequency of sensitivity to Ciprofloxacin (91%), Gentamicin (94%), Vancomycin (97%), and Erythromycin (91%) (Table 4).

**TABLE 3:** Antimicrobial susceptibility profile of isolated Gram‐negative bacteria.

	Gram‐negative bacteria (*n* = 85)		
Antibiotics	*S* ^a^ (%)	*R* ^b^ (%)	*I* ^c^ (%)
Cefuroxime	70 (82)	15 (18)	0
Ampicillin	37 (44)	48 (56)	0
Ciprofloxacin	84 (99)	0	1 (1)
Tetracycline	54 (64)	31 (36)	0
Trimethoprim–sulfamethoxazole	70 (82)	15 (18)	0
Amoxicillin/clavulanic acid	68 (80)	17 (20)	0
Gentamicin	84 (99)	1 (1)	0
Meropenem	85 (100)	0	0
Ceftazidime	55 (65)	30 (35)	0
Norfloxacin	2 (2)	83 (98)	0
Nitrofurantoin	73 (86)	12 (14)	0

^a^ Sensitive.

^b^ Resistant.

^c^ Intermediate.

**TABLE 4:** Antimicrobial susceptibility profile of isolated Gram‐positive bacteria.

	Gram‐positive bacteria (*n* = 34)		
Antibiotics	*S* ^a^ (%)	*R* ^b^ (%)	*I* ^c^ (%)
Vancomycin	33 (97)	1 (3)	0
Clindamycin	25 (74)	9 (26)	0
Ciprofloxacin	31 (91)	3 (9)	0
Tetracycline	26 (76)	8 (24)	0
Trimethoprim–sulfamethoxazole	26 (76)	3 (9)	5 (15)
Erythromycin	31 (91)	3 (9)	0
Gentamicin	32 (94)	2 (6)	0
Penicillin G	15 (44)	19 (56)	0
Oxacillin	22 (65)	12 (35)	0

^a^ Sensitive.

^b^ Resistant.

^c^ Intermediate.

### 3.4 Association between antimicrobial susceptibility profile and body part bacteria were isolated from as well as sex and origin of non‐human primates

A chi‐square test was performed to determine the relationship between the antimicrobial susceptibility profile and the body part (oral cavity and rectal region) bacteria were isolated from the origin and sex of NHPs. There was a significant association (*p* = 0.000119) between the antimicrobial susceptibility profile and body part, but not with the origin of NHPs (wild and captive), and sex of NHPs (male and female) (Table 5).

**TABLE 5:** Chi‐square test assessing association between antimicrobial susceptibility profile and body part bacteria were isolated from, origin of non‐human primate and sex of non‐human primate.

	Observed		Expected		
	*S* ^a^	*R* ^b^	*S* ^c^	*R* ^d^	*p‐*Valu*e*
**Body part**					
Oral cavity	385	117	413.4704421	88.52955787	0.000119
Rectal region	1273	238	1244.529558	266.4704421	
**Origin of non‐human primate**					
Wild non‐human primate	408	78	400.2921013	85.70789866	0.292197
Captive non‐human primate	1250	277	1257.707899	269.2921013	
**Sex of non‐human primate**					
Male	1087	222	1078.153005	230.8469945	0.277956
Female	571	133	579.8469945	124.1530055	

^a^ Observed results of sensitivity of isolated bacteria to antibiotics tested.

^b^ Observed results of resistance of isolated bacteria to antibiotics tested.

^c^ Expected results of sensitivity of isolated bacteria to antibiotics tested.

^d^ Expected results of resistance of isolated bacteria to antibiotics tested.

## 4 DISCUSSION

In this study, we aimed to characterize the oral and rectal microbiota of NHPs in Ghana and their antimicrobial susceptibility profile. Opportunistic pathogens, such as *E. coli*, *Klebsiella* spp., *Proteus* spp., *Enterobacter* spp. and *Staphylococcus* spp., were isolated from both oral and rectal swabs. *Enterobacter* spp. had the highest average phenotypic resistance to antibiotics used for AST among Gram‐negatives, whereas *Staphylococcus* spp. had the highest average phenotypic resistance to antibiotics used for AST among Gram‐positives. The bacterial isolates from the oral and rectal swabs were resistant to antibiotics, such as Norfloxacin, Ampicillin, Tetracycline, penicillin G and oxacillin.

In this study, *Staphylococcus* spp. were the dominant isolates from oral swabs, specifically, *S. haemolyticus*. *Staphylococcus* spp. have been isolated from the mucosa of NHPs in many studies (Albuquerque et al., 2020; Carvalho et al., 2014; Egbetade et al., 2020) and are important bacterial pathogens that are known to cause pneumonia, nephritis and other debilitating conditions in NHPs (Carvalho et al., 2014). All NHPs in this study had frequent contact with humans especially those that were kept as pets. Bites inflicted by NHPs usually serve as a means of dissemination of oral pathogenic bacteria (Abrahamian & Goldstein, 2011).

In our study, we isolated pathogenic enteric bacteria, such as *E. coli, Klebsiella* spp*., Proteus mirabilis*, and *Enterobacter* spp. NHPs have been reported to harbour or serve as reservoirs of enteric bacterial pathogens (Okwori et al., 2014; Parmar et al., 2013). *E. coli* was the most prevalent enteric pathogen followed by *K. pneumoniae*. This was similar to a study conducted in the United States of America where *E. coli* was the most common enteric Gram‐negative bacilli isolated from clinically healthy NHPs in captivity (Carrier et al., 2009). This may be because *E. coli*, as part of the normal flora, is one of the most common bacteria in the gastrointestinal tract of mammals. However, there are pathogenic strains of *E. coli* that are known to cause pathologic conditions, such as diarrhoea, septicaemia and other life‐threatening illnesses (Carvalho et al., 2014). Pathogenic strains of *E. coli* such as enteropathogenic *E. coli* (EPEC) have been reported in both human and NHPs and are responsible for debilitating pathologic conditions which include diarrhoea, vomiting and fever (Qi et al., 2017). Transmitted via the faecal–oral route, EPEC and other diarrhoeagenic *E. coli* are important agents of infantile diarrhoea in humans especially in developing countries such as Ghana (Carvalho et al., 2003). That we isolated *K. pneumoniae* from NHPs is of public health concern. In NHPs, *Klebsiella* spp. are important enteric pathogens responsible for debilitating pathologic conditions that result in high morbidity and mortality. There have been reports of *K. pneumoniae* outbreaks in NHPs (Carvalho et al., 2014; Kasuya et al., 2017).

AMR is a global health issue with predicted 10 million deaths annually by 2050 (Band et al., 2016; O'Neill, 2016). In Africa, the easy accessibility (over the counter) and low cost of antimicrobial agents have led to its abuse in humans and livestock (Weiss et al., 2018). Although there is a minimal use of antimicrobial agents in captive NHPs, it has been reported that wild NHPs acquire ARB through close contact with humans (Parsons et al., 2021), livestock and environmental contaminants. In this study, ARB was isolated from NHPs that live in anthropized environments and have high contact with humans. Bacterial isolates from captive NHPs that are more exposed to antibiotics had higher resistance in their antimicrobial susceptibility profile compared to wild NHPs that are less exposed (Table 5). There was an observed trend of phenotypic resistance against Norfloxacin, Ampicillin and Tetracycline among Gram‐negative bacteria (mostly *Enterobacteria*). Ampicillin and Tetracycline are broad‐spectrum antibiotics commonly used to manage a wide range of infections. Their widespread indiscriminate use has resulted in acquired resistance among many bacterial pathogens (Chopra & Roberts, 2001; Kaushik et al., 2014). Ampicillin and Tetracycline are classified as ‘critically important’ and ‘highly important’ antimicrobials by the World Health Organization (WHO) (WHO Advisory Group on Integrated Surveillance of Antimicrobial Resistance, 2018). This study also recorded that *Enterobacter* spp. had the highest average phenotypic resistance among Gram‐negative bacterial isolates. This is quite alarming because there is currently an emergence of ARB among *Enterobacter* spp. and other members of the family Enterobacteriaceae in humans (Band et al., 2016). *Enterobacter* spp. is one of the most common Carbapenem‐resistant Enterobacteriaceae with multidrug resistance to last resort Carbapenems in humans (Annavajhala et al., 2019). *Enterobacter* spp. are clinically important pathogens associated with multidrug‐resistant nosocomial infections in humans (Band et al., 2016).

Among Gram‐positive bacterial isolates, there was a trend of resistance against penicillin G and oxacillin. This is in tandem with a study in Nigeria that reported resistance against Penicillin antibiotics among bacterial isolates from oral and rectal swabs of captive NHPs (Egbetade et al., 2020). The resistance to Penicillin antibiotics may be due to the inappropriate use of these broad‐spectrum antibiotics in humans and in animal husbandry leading to the selection for resistant bacteria (Yevutsey et al., 2017). Penicillin G is classified as ‘highly important’ under the WHO antimicrobial classification (WHO Advisory Group on Integrated Surveillance of Antimicrobial Resistance, 2018). Resistance to penicillin G is therefore significant for public health. *Staphylococcus* spp. recorded the highest average phenotypic resistance to antibiotics used for AST among Gram‐positive bacterial isolates. In this study, *S. haemolyticus* was the most occurring of the resistant staphylococcal species. *S. haemolyticus* is an opportunistic pathogen known to cause nosocomial infections as well as infections in several body systems in humans (Eltwisy et al., 2020). Therefore, the isolation of resistant staphylococcal species in the NHPs is important for public health.

We found a significant association between the body part (oral and rectal) from which the bacteria were isolated from and the antimicrobial susceptibility profile. This was expected and in tandem with studies in humans where AMR was associated with enteric bacterial composition (Covington & Parmer, 2017; Isaac et al., 2017). Exposure to antimicrobials, even at low levels, in the environment leads to the selection for AMR bacteria in microflora‐rich environments such as the oral cavity and the gastrointestinal tract (Kim et al., 2020). The oral and rectal microbiota of NHPs have been associated with the transmission of pathogenic AMR bacteria through bites and the faecal–oral route (Abrahamian & Goldstein, 2011; Egbetade et al., 2020; Nguema et al., 2021; Sobreira et al., 2019).

We note here that the culture‐based method of identifying bacteria limited the isolation of bacteria to only isolates that could grow aerobically on artificial culture media which underestimated the total diversity and richness of the bacterial community. Future studies should, therefore, consider a combination of culture‐based methods and molecular methods such as metagenomic sequencing to circumvent the limitations imposed by culture‐dependent methods on the diversity of the bacterial community of NHPs.

## 5 CONCLUSION

Despite some limitations, the results of our study revealed that apparently healthy NHPs in Ghana harbour zoonotic pathogenic bacteria. It was also discovered that NHP‐associated bacteria were resistant to commonly used antibiotics in humans and livestock. This study demonstrates the necessity for surveillance systems for zoonotic pathogens and AMR among NHPs within the One Health concept. This is crucial in the prevention of the dissemination of zoonotic and antimicrobial‐resistant bacteria among humans and NHPs.

## AUTHOR CONTRIBUTIONS

*Data curation; formal analysis; investigation; writing – original draft*: Eugene Adade. *Data curation; formal analysis; investigation; writing – review and editing*: Patrick Ofori Tawiah. *Conceptualization; funding acquisition; writing – review and editing*: Christian Roos. *Methodology; formal analysis; investigation; writing – review and editing*: Idrissa Shomari Chuma, Clara Clavery Lubinza and Sayoki Godfrey Mrinde Mfinanga. *Conceptualization; funding acquisition; methodology; project administration; supervision; writing – review and editing*: Sascha Knauf. *Conceptualization; funding acquisition; methodology; project administration; supervision; validation; writing – review and editing*: Augustina Angelina Sylverken.

## CONFLICT OF INTEREST STATEMENT

The authors declare that they have no conflicts of interest.

## FUNDING INFORMATION

The German Research Foundation, Funding number KN, AAS 1097/3‐2 and RO 3055/2‐2.

### ETHICS STATEMENT

The authors confirm that the ethical policies of the journal as noted on the journal's author guidelines page have been adhered to. Non‐human primate samples were taken with the permission of the Wildlife Division of the Forestry Commission of Ghana (Reference No. 0095189, project code: 01/01/2018) and according to their approved guidelines. Good Veterinary Practice rules were applied to all procedures when NHPs were handled for sampling. All procedures were conducted by a licensed veterinarian.

### PEER REVIEW

The peer review history for this article is available at https://publons.com/publon/10.1002/vms3.1271.

## Data Availability

The data that support the findings of this study are fully displayed in this article.
